# Avoidance and Potential Remedy Solutions of Chimeras in Reconstructing the Phylogeny of Aphids Using the 16S rRNA Gene of *Buchnera*: A Case in Lachninae (Hemiptera)

**DOI:** 10.3390/ijms160920152

**Published:** 2015-08-25

**Authors:** Rui Chen, Zhe Wang, Jing Chen, Ge-Xia Qiao

**Affiliations:** 1 Key Laboratory of Zoological Systematics and Evolution, Institute of Zoology, Chinese Academy of Sciences, Beijing 100101, China; E-Mails: chrui11@live.cn (R.C.); wzhe1226@126.com (Z.W.); chenjing@ioz.ac.cn (J.C.); 2 College of Life Sciences, University of Chinese Academy of Sciences, Beijing 100049, China; 3 Institute of Plant Protection, Liaoning Academy of Agricultural Sciences, Shenyang 110161, China

**Keywords:** chimera, 16S rRNA gene, *Buchnera*, phylogeny, aphids, Lachninae

## Abstract

It is known that PCR amplification of highly homologous genes from complex DNA mixtures can generate a significant proportion of chimeric sequences. The 16S rRNA gene is not only widely used in estimating the species diversity of endosymbionts in aphids but also used to explore the co-diversification of aphids and their endosymbionts. Thus, chimeric sequences may lead to the discovery of non-existent endosymbiont species and mislead *Buchnera*-based phylogenetic analysis that lead to false conclusions. In this study, a high probability (6.49%) of chimeric sequence occurrence was found in the amplified 16S rRNA gene sequences of endosymbionts from aphid species in the subfamily Lachninae. These chimeras are hybrid products of multiple parent sequences from the dominant species of endosymbionts in each corresponding host. It is difficult to identify the chimeric sequences of a new or unidentified species due to the high variability of their main parent, *Buchnera aphidicola*, and because the chimeric sequences can confuse the phylogenetic analysis of 16S rRNA gene sequences. These chimeras present a challenge to *Buchnera-*based phylogenetic research in aphids. Thus, our study strongly suggests that using appropriate methods to detect chimeric 16S rRNA sequences may avoid some false conclusions in endosymbiont-based aphid research.

## 1. Introduction

Aphidina live in association with a diverse assemblage of heritable intra-cellular bacterial endosymbionts [1,2,3]. The endosymbionts are categorized as either primary or secondary. Primary endosymbionts are essential for the survival of their aphid hosts [4,5,6,7]. Almost all aphids have the primary endosymbiont *Buchnera aphidicola* (Gammaproteobacteria: Enterobacteriales: Enterobacteriaceae), which supplies essential nutrients lacking in aphid diet [8,9,10,11,12,13]. In addition, aphids can have a series of secondary endosymbionts, such as *Regiella insecticola* (Enterobacteriaceae), *Hamiltonella defensa* (Enterobacteriaceae), *Serratia symbiotica* (Enterobacteriaceae) [14], *Wolbachia pipientis* (Alphaproteobacteria: Rickettsiales) [15], and a *Sodalis*-like symbiont (Enterobacteriaceae) [16]. These benefit their aphid hosts by providing protection against parasitoids, pathogens, or thermal stress, though they are generally not required for host development and reproduction [17]. These secondary symbionts inhabit a variety of tissues, such as sheath cells, hemolymph, and bacteriocytes [14].

Due to the biological importance of the endosymbionts to the aphid hosts, the relationship between endosymbionts and aphids has become a hotspot of research. Many researchers have focused on the evolutionary relationship between the aphids and endosymbionts [16,18,19,20,21,22,23,24]. By comparing the phylogeny of *Buchnera* based on the 16S rRNA gene and the phylogeny of aphids based on morphological features and the 16S rRNA gene, previous studies have indicated that *Buchnera* is completely concordantly evolved with its aphid hosts [12,18,25]. Based on the ages of aphid fossils, biogeographical events, and the estimated substitution rates of the 16S rRNA gene, the minimum age of *Buchnera* association was estimated at 160–280 million years [18]. In the phylogenetic research of aphids, *Buchnera* markers, especially 16S rRNA gene was widely used as the third genome due to the parallel evolution with aphid hosts, thus, is a very important marker to reconstruct the phylogenetic relationship in different taxonomic levels in aphids [13,16,18,19,20,21,22,24].

The 16S ribosomal RNA gene (hereafter 16S) has been widely used in symbiont-based research as an important marker for bacterial taxonomic and phylogenetic studies. Although sequence analysis of 16S was widely used to evaluate the diversity and identities of bacterial species in insect hosts [16,26,27], many studies have reported that 16S amplicon sequencing using the PCR method can misrepresent the abundance of the microbial population because of the presence of chimeras [28,29,30]. Chimeric sequences are usually PCR artifacts. They are believed to occur when a prematurely terminated amplicon reanneals to foreign bacterial DNA included in the same extraction and is then copied to completion in the subsequent PCR cycles. PCR-generated chimeric sequences usually consist of two phylogenetically distinct parent sequences, as other intra-cellular bacteria are phylogenetically distinct from *Buchnera*. The chimeras will lead to inaccurate clustering in phylogenetic studies, and also artificially increase estimates of diversity in culture-independent surveys of microbial communities because they suggest the presence of nonexistent organisms. Prior analyses have indicated that 3.8% of the 16S sequences from the Bacteroidetes phylum (2739 sequences) were apparently chimeric [31]. In aphids, multiple endosymbionts, such as *Buchnera* and other facultative symbionts, reside in one individual, creating an opportunity for producing chimeras. Furthermore, due to multiple infections and the universality of primers of 16S, cloning sequencing rather than direct sequencing was widely used in this kind of study [16,32]. Treating the chimeric sequences as normal sequences would result in ambiguous or even false results. However, there are so far no reports of chimeric sequences in endosymbiont-based research on Aphidina.

Here, we selected the Aphididae subfamily Lachninae for a case study. The Lachninae species are known to have a high incidence of facultative symbiont infection [16], which makes Lachninae a perfect object to study the influences of chimeric sequences in endosymbiont-based aphid research. We survey the presence of chimeras in the amplification of 16S sequences of endosymbionts with universal primers. And more importantly, we evaluated the effect of these chimeras on the phylogenetic reconstruction of aphids and endosymbionts.

## 2. Results

### 2.1. Prevalence and Types of Pure Sequences and Chimeras

**Figure 1:**
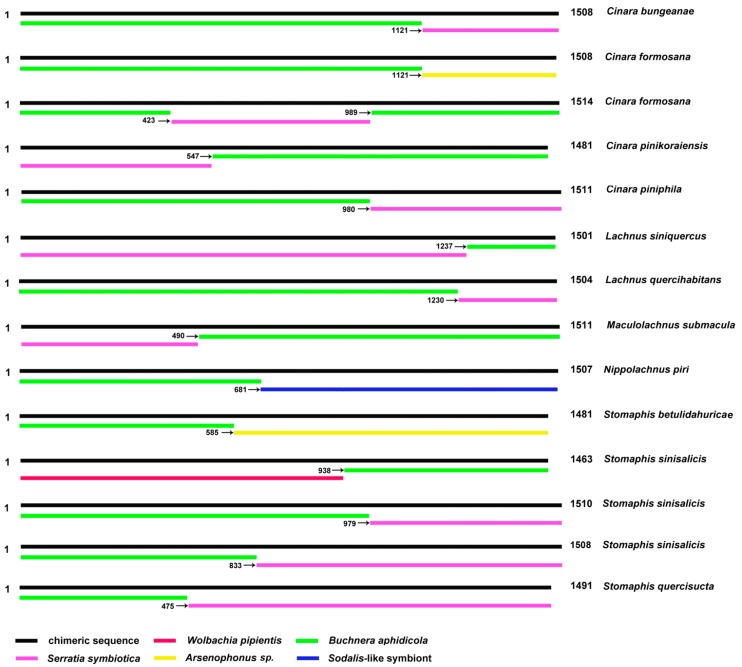
The types of chimeric sequences. Number at right end of bar giving length of sequence; number associated with arrow gives the sequence position where the sequence portion to the right of it starts.

The *Buchnera* 16S gene sequences from 12 Lachnine species were amplified and sequenced. Chromatograms from direct sequencing had a single peak in all nucleotide positions in two samples (Supplementary File S1), but miscellaneous peaks in 10 samples (see Supplementary File 2. These two sequences were proved to be *Buchnera* 16s rRNA gene sequence through BLAST tool at NCBI and manually checking. Whereas the other 10 did not bear any semblance to known sequences. In other words, only 16.7% (2/12) effective sequences were obtained by direct sequencing. In cloning experiments of our study, 185 positive clones were selected to be sequenced; 99 were pure *Buchnera* sequences, 58 were *Serratia symbiotica* sequences, one was *Wolbachia pipientis* sequence, five were *Arsenophonus* sp. sequences, nine were *Sodalis*-like symbiont sequences, one was a *Regiella insecticola* sequence, and 12 were suspected chimeric sequences. Examination of these suspected chimeric sequences with the grammar DECIPHER [33] and UCHIME [34] both indicated that these sequences were not chimeras. Then we manually aligned these sequences with the 16S sequences of endosymbionts in aphids. We found that these suspected chimeric sequences were chimeras indeed. The occurrence frequency of chimeras was 6.49% across the Lachninae. The parental sequences of these chimeric sequences were 16S from *Buchnera*, *Serratia symbiotica*, *Wolbachia pipientis*, *Arsenophonus* sp., and a kind of *Sodalis*-like symbiont; mitochondrial 16S was not involved. According to the position of the parental sequence in the chimeric sequence, three types of chimeric sequences were identified, namely “BA + oe”, “oe + BA”, and “BA + oe + BA” (“BA” = *Buchnera aphidicola*, “oe” = “other endosymbiont” (Figure 1 and Figure S1). The positions of the chimera breakpoints were at different conservative regions of the 16S sequences, which are most likely to be similar in phylogenetically remote bacteria.

### 2.2. Phylogenetic Analysis of the Two Data Sets

**Figure 2:**
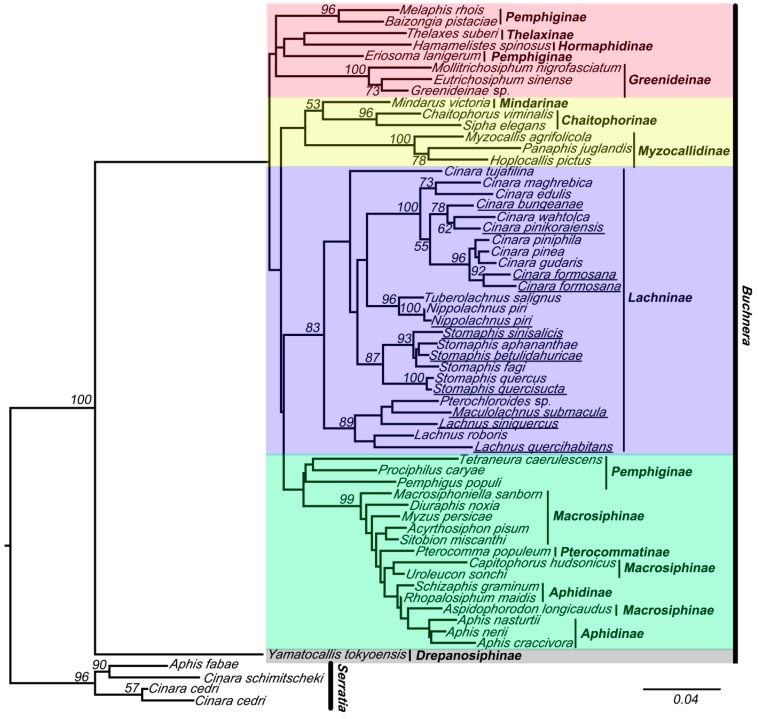
The maximum likelihood (ML) phylogenetic tree inferred from data-set I (without chimeric sequences). *Buchnera* sequences are represented by the names of their host species. The sequences obtained from this study are underlined. The nodes are marked by their ML bootstrap values. The bar represents 4% of sequence change with regard to the likelihood distance. The underlined species are with chimeric sequences.

**Figure 3:**
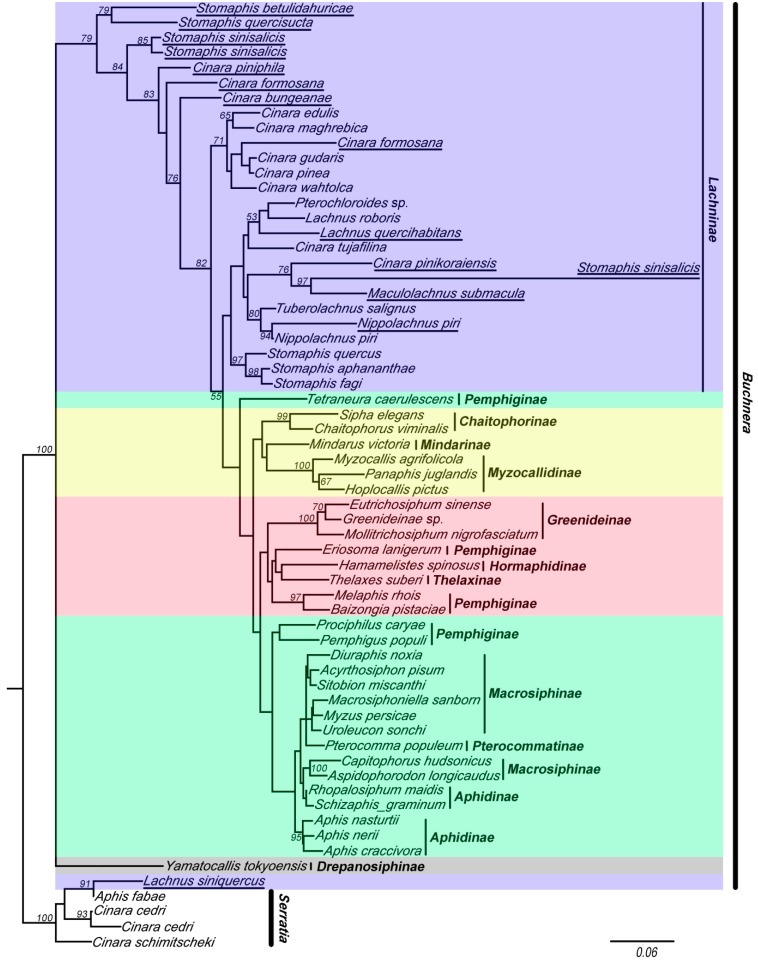
The maximum likelihood (ML) phylogenetic tree inferred from data-set II (with chimeric sequences, violet clade). *Buchnera* sequences are represented by the names of their host species. The chimeric sequences obtained from this study are underlined. The nodes are marked by their ML bootstrap values. The bar represents a 6% sequence change with regard to the likelihood distance. The underlined species are with chimeric sequences.

The results of the analyses of these two data sets indicated that the 16S sequences of *Buchnera* clustered into five clades (red, yellow, violet, green, and gray clade in Figure 2 and Figure 3). Tree topologies were very different for data-set II (with chimeric sequences) (Figure 3) and data-set I (Figure 2). The violet clade (= Lachninae) is monophyletic and forms the sister group of the green clade in Figure 2, while it is a “basal” paraphyletic assemblage in Figure 3. The species *Tetraneura caerulescens* (non-chimeric sequence) falls into the green clade in Figure 2, while in Figure 3 it is remote from this clade. All chimeric sequences fell into the in group, except for the chimeric sequence from *Lachnus siniquercus* (Figure 3). According to the results of the SH test, the difference between the phylogenetic trees constructed by data-sets I and II was significant because the *p* value was <0.05. Thus, chimeric sequences can confuse the phylogenetic structure of *Buchnera* based on strict host correlation. We also emphasize that the ML bootstrap values are higher in data-set I than in data-set II.

## 3. Discussion

### 3.1. Composition of Chimeras in Aphid Endosymbionts

A high probability (6.49%) of chimeric sequence occurrence was found when amplifying the 16S rRNA gene sequences of endosymbionts from Lachninae. Most chimeric sequences were formed with the aphid primary symbiont *Buchnera* as well as the secondary symbiont *Serratia symbiotica*, which was found in many Lachninae [16,35] (Figure 1). *S. symbiotica* may be beneficial to hosts due to their ability to supplement nutrition and to thus compensate for the inadequate provision of nutrients by *Buchnera* [16]. Moreover, *Arsenophonus* and *Sodalis*-like symbionts were found more likely to infect the genera *Stomaphis* and *Nippolachnus* [16]. Accordingly, the chimeric sequences from the two species *Stomaphis sinisalicis* and *Nippolachnus piri* included fragments from *Buchnera*, *Arsenophonus*, and *Sodalis*. In addition, *Arsenophonus* was found in *Cinara formosana* in our study, and a chimera composed of *Buchnera* and *Arsenophonus*was also found in that aphid species*. Wolbachia* is widely distributed in insects and was found in some species of Lachninae but not in *Stomaphis* [15]. In our study, *Wolbachia* was found in *Stomaphis sinisalicis* along with a chimeric sequence including *Buchnera* and *Wolbachia* fragments. All this supports that chimeras created from the predominant species of endosymbionts can in different aphid taxa form during PCR.

According to Hugenholtz and Huber [28], chimeras are commonly formed from closely related parental sequences due to sequence similarity. Here, endosymbionts of parental sequences such as *Buchnera*, *Serratia*, *Arsenophonus*, and *Sodalis* all come from Gammaproteobacteria [14], which has ten conserved regions in the 16S gene sequence [36]. It is because of the existence of these conserved regions, coupled with two or more dominant symbionts living in one aphid individual, so it is likely that the obtained sequences were chimeras.

### 3.2. Disguised Chimeric Sequences in Buchnera-Based Research

The 16S rRNA gene is widely used for bacterial taxonomic and phylogenetic studies because its divergence is large enough to discriminate between varieties of bacteria [36]. For *Buchnera*, the divergences of the 16S rRNA sequences among different aphid host taxa are large enough for it to be used to reconstruct the phylogenetic relationships of the corresponding host taxa [16,20,23]. Some intraspecific divergence values of *Buchnera* 16S sequences from different aphid species are even larger than the interspecific divergence values of different bacteria. For example, the highest divergence values of *Buchnera* from different aphid species in our studies reach 12.8%, while bacterial lineages with more than 3% divergence of 16S rRNA are recognized as distinct OTUs [37,38]. It is difficult to identify chimeric sequences from a new or unidentified aphid species as such due to the high variation of 16S in *Buchnera*. When a new sequence of *Buchnera* is encountered, a search of the BLAST databases in NCBI [39] and the reconstruction of the phylogenetic tree can help to identify the species of the new sequence. However, when a BLAST search is done for a chimeric sequence, the result might indicate that the chimeric sequences are most similar to one or more sequences that may come from only one of the parent sequences identified by other research, such as *Buchnera* (Figure S2). Therefore, the chimeras can be misinterpreted as representing *Buchnera* even though they are actually from a novel aphid species.

Likewise, when using phylogenetic reconstruction to address the sequences, chimeric sequences are usually clustered into one clade with pure *Buchnera* sequences (Figure 2), and chimeric sequences can be put into any position in phylogenetic tree. Moreover, in some taxa, such as *Nippolachnus piri*, the chimeric sequence and the normal sequence can cluster together. Thus, chimeric sequences are difficult to identify by conventional detection methods. Programs used for detecting chimeras, such as CHIMERA_CHECK in RDP [40], found the chimeras by determining whether fragments of two independent database entries had a higher overall similarity to the query sequence than a single, full-length database entry [41,42]. Unfortunately, if the parent sequences are unknown, this method is similarly unhelpful. Thus, understanding the composition of the dominant symbionts in different aphid taxa is a prerequisite to finding the parent sequences.

In our study, we used the Find Chimeras function in DECIPHER package [43] and UCHIME to find the chimeras. The software programs DECIPHER and UCHIME are widely used to checking chimera. The DECIPHER package was high-efficiency. When evaluated with the data set of simple two-parent chimeras, ss_DECIPHER and fs_DECIPHER detected 88% and 75% of the chimeras, while Uchime, ChimeraSlayer, and WigeoN detected 73%, 56%, and 47%, respectively [33]. Different from DECIPHER, UCHIME is a chimera finding algorithm that uses a premise to detect the chimeras that sometimes DECIPHER did not [34]. The results of DECIPHER and UCHIME both indicated that the suspected chimeric sequences obtained in our study were not chimeras. Then we manually aligned these sequences with the 16S sequences of endosymbionts in aphids. We found that these suspected chimeric sequences were chimeras. In addition, based on the phylogenetic tree, we found that the chimeras have two traits. Firstly, the chimeras in the *Buchnera* tree showed a tendency to be dragged far to the base of the tree (for example, chimeras from *Stomaphis*, Figure 3), because the chimeric sequences whose partner, for example *Serratia symbiotica*, was used as outgroup in our study. Secondly, chimeras should be placed further up in the tree but on very long branches (for example, chimera from *Stomaphis sinisalicis*).

Moreover, there were some strange aspects in the tree of data-set II (with chimeras) (Figure 3). For example, *Cinara formosana* (chimera without *Serratia symbiotica*, Figure 1) was closer to the base than *Cinara bungeaneae* (chimera with *Serratia symbiotica*); *Stomaphis betulidahuricae* (chimera without *Serratia*, Figure 1) and *Stomaphis quercisucta* (chimera with *Serratia symbiotica*) form a clade, and especially both have similar branch lengths compared to outgroup *Serratia symbiotica*. The reason which caused this phenomenon may be the higher difference within *Buchnera*. According to our analysis, the genetic distance within *Buchnera* was from 0.2% to 12.8%; the genetic distance between *Buchnera* and other symbionts was: 14.2% (*Buchnera* and *Serratia symbiotica*), 12.4% (*Buchnera* and *Sodalis*-like symbiont), 15.0% (*Buchnera* and *Arsenophonus* sp.), and 26.0% (*Buchnera* and *Wolbachia*). So the branch length of chimeras which formed with *Buchnera* and *Serratia symbiotica* was similar to the outgroups, and the chimera which formed with *Buchnera* and *Wolbachia* formed a long branch clade.

### 3.3. Effects of Chimeric Sequences on Buchnera-Based Research

Our results showed that chimeric sequences affect the 16S phylogeny of *Buchnera*. The comparisons in our study showed that the chimeric sequences confused the phylogenetic analysis of the 16S rRNA gene sequences. Although only part of these chimeric sequences came from *Buchnera* and the lengths of the *Buchnera* sequences within the chimeric sequences were less than half of the total sequence, these chimeras were still clustered with the *Buchnera* sequences, such as with the chimera from *Stomaphis sinisalicis* (Figure S1). The addition of these chimeras changed the relationship of the normal sequences and led to incorrect results (Figure 2 and Figure 3). With the parallel evolution of *Buchnera* and its aphid hosts as demonstrated in Aphidoidea [24], many researchers focus on reconstructing the phylogeny of aphids based on the genes of *Buchnera*. The 16S rRNA gene was necessary in related studies. However, the chimeras will challenge *Buchnera-*based aphid phylogenetic research. The changes to the topology of the phylogenetic tree caused by chimeras may mislead the analysis and may lead to false conclusions.

In a recent report, the *gnd* gene of *Buchnera* combined with the mitochondrial COI gene was selected as an efficient aphid barcode [44]. It puts forward a new idea that using the gene of the endosymbiont to identify host species. In theory, the 16S rRNA gene of *Buchnera* is also a suitable barcode marker to identify aphid due to its high-divergence among diverse host taxa. However, the high frequency of the occurrence of chimera seriously affects the utility of the 16S rRNA gene as a barcode for identifying the species. Our study strongly suggests that we should pay more attention to abnormal sequences in the alignment when the 16S gene is used for endosymbiont-based aphid research; and use appropriate methods to detect chimeric 16S rRNA sequences can avoid some false conclusions in endosymbiont-based aphid research. Thus, we could reduce the effect of chimeras in the following ways: (1) improve the PCR conditions to decrease the chimeras, such as designing specific primers for different species in hypervariable regions of the 16S rRNA gene; using touch down PCR to improve the specificity of PCR amplification; and using Hi Fi Taq polymerase to ensure the accuracy of the PCR amplification, although the chimeras cannot be eliminated entirely in experiments [45]; (2) defining a selection of confirmed pure 16S sequences of the various bacterial taxa at GenBank (or in a separate tool), and then dividing new 16S sequences within the conserved regions, and blast the portions separately against the pure sequences.

## 4. Experimental Section

### 4.1. Samples, DNA Extraction, PCR, Cloning, and Sequencing

We sampled 12 species from Aphididae-Lachninae (Table 1). Three to five individuals per sample were used as slide-mounted specimens for morphological identification. All samples were stored in 95% or 100% ethanol and deposited in the National Zoological Museum of China, Institute of Zoology, Chinese Academy of Sciences, Beijing, China.

DNA extraction was performed with a single aphid from each sample using a DNeasy kit (Qiagen, Frankfurt, Germany). PCR was used to amplify the 16S rRNA gene sequence using the primer pair 16SF (5′-AGAGTTTGATCATGGCTCAGATTG-3′) and 16SR (5′-TACCTTGTTACGACTTCACCCCAG-3′), which was designed specifically for *Buchnera* [46], and is widely used in studies of aphid endosymbionts [16,20,23]. PCR amplification was performed in a 30 μL reaction volume consisting of 3.0 μL 10× PCR buffer, 2.4 μL dNTPs (10 mM each), 20 μL dd H_2_O, 0.6 μL of each 10 μM forward and reverse primers, and one unit of *Taq*DNA polymerase. Every PCR included a negative control (double-distilled water instead of DNA). The PCR conditions were as follows: 95 °C for 5 min; 35 cycles consisting of denaturation at 95 °C for 1 min, 65 °C for 0.5 min, and extension at 72 °C for 2 min; and a final extension period at 72 °C for 10 min.

Every PCR product was purified using a DNA Fragment Purification kit (TransGen, Beijing, China). Then two methods were used to obtain the sequences of every PCR products: directly sequencing and cloning. In the process of directly sequencing part of every purified PCR product was put in the sequencer and sequenced. During cloning experiments, the every other PCR purified product was ligated into the plasmid vector pMD19-T (TaKaRa, Dalian, China), and at least 20 clones from each product were sequenced on an ABI 3730 automated sequencer. Both strands of the plasmids were sequenced using universal primers (M13+, M13**−**) with forward and reverse reads. All sequences obtained from this study were deposited in GenBank (accession numbers in Table 1). Chimeric sequences are shown in Supplementary File S3.

### 4.2. Sequence Analysis

The sequences obtained were assembled using SeqManII of Lasergene v5.0 (DNASTAR, Madison, WI, USA) and were manually verified in DNAMAN v5.2.2.

Chimeric sequences were checked using the DECIPHER package through the use of the Find Chimeras function [43] and UCHIME in reference mode [34].

To estimate the effect of these chimeras on the phylogenetic analysis of *Buchnera*, two data sets were selected for analysis. Data-set I was made up of 61 normal 16S sequences of *Buchnera*, including 45 sequences downloaded from GenBank [16,20,24,25,47,48,49,50,51,52,53] (Table S1), 12 sequences from this study (Table 1), and four sequences of *Serratia symbiotica* (Table S2) that were defined as the outgroup [54]. The aphid hosts of *Buchnera* in data-set I represented 12 subfamilies of Aphididae. Most of the data in data-set II was the same as in data-set I except that in many Lachninae normal sequences obtained from our study were replaced by authentic chimeras that we identified after PCR (Supplementary Files S1 and S2). Each data set was aligned using ClustalX v1.8.3 with the default settings [55]. Shimodaira-Hasegawa (SH) tests of topology [56] were carried out using PAUP 4b10 to assess the level of incongruence between these two data sets.

The phylogenetic analysis was conducted using the maximum likelihood (ML) method. The ML analyses were performed in RAxML7.2.8, using a heuristic search with the GTRCAT model and bootstrapped with 1000 replicates [57].

**Table 1:** The detailed collection information and GenBank accession numbers of endosymbionts of Lachninae species.

Species	Location (China)	Collection Date	No. Voucher	GB. Number *Buchnera*	GB. Number Other Symbionts
*Cinara bungeanae* (Zhang *et al*., 1993) [58]	Beijing	30.iv.2005	16107	KF751194	KF751206
*Cinara formosana* (Takahashi, 1924) [59]	Fujian: Wuyi Mountains	21.x.2005	18072	KF751197	KF751209
*Cinara formosana* (Takahashi, 1924) [59]	Yunnan: Lijiang City	27.iv.2006	18216	KF751198	JN990929
*Cinara pinikoraiensis* (Zhang, 1989) [60]	Heilongjiang: Yichun City	10.viii.2005	17836	KF751196	KF751208
*Cinara piniphila* (Ratzeburg, 1844) [61]	Inner Mongolia: HulunBuir	13.viii.2004	15921	KF751193	KF751205
*Lachnus quercihabitans* (Takahashi, 1924) [59]	Guangxi: Lingui County	03.xi.2010	26064	KF751202	KF751214
*Lachnus siniquercus* (Zhang, 1982) [62]	Guizhou: Leigong Mountain	04.vi.2005	16278	KF751195	KF751207
*Maculolachnus submacula* (Walker, 1848) [63]	Xinjiang: Nilka County	05.ix.2002	13796	KF751192	KF751204
*Nippolachnus piri* (Matsumura, 1917) [64]	Anhui: Yuexi County	21.vii.2007	20199	KF751201	KF751213
*Stomaphis betulidahuricae* (Zhang *et al*., 1999) [65]	Beijing	23.vii.2006	19448	KF75200	KF751212
*Stomaphis quercisucta* (Qiao *et al*., 1999) [65]	Beijing	29.viii.2009	Y8896	KF751203	KF751215
*Stomaphis sinisalicis* (Zhang *et al*., 1982) [62]	Beijing	15.vi.2006	19106	KF751199	KF751210/KF751211

## 5. Conclusions

Our research indicated that chimeric sequences were usually obtained when amplifying 16S rRNA gene sequences of endosymbionts from aphid species. It is difficult to identify the chimeric sequences from a new or unidentified species. These chimeric sequences always confuse the phylogenetic analysis of 16S rRNA gene sequences. So we must pay attention to the chimera when studying endosymbionts in aphids, and identify the chimera according to the ways proposed in this article.

## Supplementary Materials

Supplementary materials can be found at http://www.mdpi.com/1422-0067/16/09/20152/s1.
